# “Allergic bronchopulmonary aspergillosis misdiagnosed as smear negative pulmonary tuberculosis; a retrospective study from Pakistan”

**DOI:** 10.1016/j.amsu.2021.103045

**Published:** 2021-11-11

**Authors:** Nousheen Iqbal, Muhammad Dawood Amir Sheikh, Kauser Jabeen, Safia Awan, Muhammad Irfan

**Affiliations:** a Section of Pulmonary and Critical Care Medicine, Department of Medicine, Aga Khan University, Karachi, Pakistan; b Jinnah Medical and Dental College, Karachi, Pakistan; c Aga Khan University, Hospital, Karachi, Pakistan; d Department of Pathology and Laboratory Medicine, Aga Khan University, Karachi, Pakistan; e Aga Khan University, Karachi, Pakistan

**Keywords:** Smear negative tuberculosis, Allergic bronchopulmonary aspergillosis, Asthma, Misdiagnosis

## Abstract

**Background:**

Undiagnosed allergic bronchopulmonary aspergillosis (ABPA) can lead to chronic persistent symptoms. In country like Pakistan where tuberculosis (TB) is endemic, a significant proportion of ABPA patients are misdiagnosed as smear negative TB before reaching a diagnosis of ABPA due to chronicity of symptoms.This lead to empiric use of ATT(Anti-tuberculous therapy) and delay in primary diagnosis. The aim of the study is to determine such proportion of ABPA patients.

**Material and methods:**

This retrospective study was conducted at the outpatient pulmonology clinic of a tertiary care hospital in Karachi, Pakistan from January 2017 to December 2018. Xpert MTB/Rif, TB smear and culture were performed in all patients to rule out active TB.

**Results:**

A total 167 of ABPA patients were included. Mean age of the patients was 41.9 ± 13.0 years, 91(54.5%) were female and 71 (42.5%) patients had received ATT in past. Out of these 71 patients, 63 (88.7%) patients were diagnosed as smear negative TB and received empiric ATT. Among 63 patients, 52 (82.5%) patient had received ATT once, 8 (12.6%) twice and 3 (4.7%) patients had received empiric ATT thrice. Of these 27 (16.16%) patients had already developed long term complications at the time of diagnosis of ABPA and 17 (62.96%) patients were in empiric TB treatment group.

**Conclusion:**

Patient with ABPA frequently received empiric ATT as smear negative TB in high TB burden country. This results in over diagnosis of TB and unnecessary use of global resource. When Gene Xpert negative alternate diagnosis should be considered.

## Highlights

• Allergic bronchopulmonary aspergillosis (ABPA) can lead to chronic persistent symptoms.
• In country like Pakistan where tuberculosis (TB) is endemic, a significant proportion of ABPA patients are misdiagnosed as smear negative TB before reaching a diagnosis of ABPA due to chronicity of symptoms which leads to empiric use of ATT.
• So the aim of the study is to determine such proportion of ABPA patients in country where TB is endemic.

## 1 Introduction

Allergic bronchopulmonary aspergillosis (ABPA) occurs due to hypersensitivity reactions in response to colonization of the tracheobronchial tree by *Aspergillus* spp [1]. Around 1–2% patients with asthma and 2–15% patients with cystic fibrosis are estimated to have concomitant ABPA [2]. Out of the reported 510,000 new cases of TB from Pakistan, almost 112,948 are smear negative for acid fast bacillus (AFB) [3]. Due to the high prevalence of TB in Pakistan, it is common to misdiagnose ABPA patients as smear negative TB with prolonged treatment with anti-tuberculous therapy (ATT) [4].

ABPA and TB share certain features that make it difficult to distinguish between these conditions. Cough, fever and hemoptysis are the presenting complaints in both entities. According to a study, almost 31% of patients with ABPA had hemoptysis as primary complaint; [5]. Similarly, a study on TB revealed that hemoptysis was a symptom in about 79.2% of patients [6]. Bilateral rhonchi, crackles and wheezes are common examination findings in both the diseases. Both entities can present with bilateral infiltrates and bronchiectasis changes.

A delayed diagnosis of ABPA can predispose patients to a number of complications including bronchiectasis, pulmonary fibrosis, respiratory failure and progressive lung damage eventually leading to death [7]. Therefor a misdiagnosis not only exposes a patient unnecessarily to ATT but also leads to a number of ABPA associated complications. Since Pakistan have high burden of TB majority of ABPA patients before reaching a diagnosis ended up with empiric ATT even they are GeneXpert negative. In this study we have highlighted these patients who were treated as smear negative TB with no clinical and radiological improvement and later turned out to be ABPA.

## 2 Material and Methods

This was a retrospective study conducted on patients diagnosed with ABPA in the outpatient adult pulmonary clinic of Aga Khan University Hospital, (AKUH) Karachi, Pakistan from January 2017 to December 2018. The study was exempted by the Ethical Review Committee of Aga Khan University Hospital. ABPA patients were identified through outpatient pulmonary clinic database. The diagnostic criteria for ABPA was adopted from ISHAM 2013 [8]. Inclusion criteria included: **1)** Age ≥18 years with underlying asthma, **2)** Serum IgE level ≥1000 IU/ml, **3)** Peripheral eosinophilia ≥500 cells/μL in steroid naïve patients, **4)** Radiographic pulmonary opacities consistent with ABPA, **5)** A positive type I *Aspergillus* skin test (immediate cutaneous hypersensitivity to *Aspergillus* antigen), **6)** Active TB and other infections were ruled out by sputum microscopy, culture and Xpert MTB/Rif. ABPA was classified into five stages, stage 1 patients with acute flare, stage 2 patients who underwent remission, stage 3 patients with recurrent exacerbations, stage 4 patients who were steroids dependent and stage 5 patients with advanced fibrotic lung disease. Data was collected on predesigned proforma which included demographics, co-morbid conditions, presenting clinical symptoms, smoking status, serum IgE and eosinophilia levels, chest X ray findings, status, duration and frequency of ATT and complications developed by these patients. Patients with an incomplete record and with evidence of active TB were excluded from this study.

## 3 Statistical analysis

SPSS V.19.0 was used for data analysis. Descriptive statistics were done, and results are presented as mean and standard deviation for continuous variable and number and percentages for categorical variables.

## 4 Results

A total of 167 patients with a known diagnosis of ABPA were selected for the study. A total 167 of ABPA patients were included. Mean age of the patients was 41.9 ± 13.0 years, 91(54.5%) were female and 71 (42.5%) patients had received ATT in past. Out of these 71 patients, 63 (88.7%) patients were misdiagnosed as smear negative TB and received empiric ATT before diagnosing ABPA. The remaining 8 patients were excluded from the study due to smear positive TB. These 167 ABPA patients were divided into two groups those who received empiric ATT, misdiagnosed as smear negative TB (63 patients) and who did not receive ATT (96 patients).

Both groups reported symptoms of weight loss, dyspnea, wheezing, chest pain, sputum pellets, cough and fever of variable duration. Cough and dyspnea were the most common symptoms in both groups. However, hemoptysis was the only variable that was found to be statistically significant in the smear negative TB group. Chest X-ray findings such as consolidations, nodules, cavitation, fibrosis and infiltrates were also found to be present in both groups. Pulmonary nodules were found to be the most common chest X-ray finding in both the smear negative ATT group and the non-ATT group (Table 1). Out of the 63 patients, 52 (82.5%) patients had received ATT once, 8 (12.6%) patients received ATT twice and, 3 (4.7%) patients had received ATT therapy for a total of three times before reaching the diagnosis of ABPA.

**Table 1:** Clinical and radiological features of ABPA patients divided in two groups based on empirical ATT.

	ABPA(misdiagnosed) treated as Smear negative TB; n = 63(%)	ABPA without TB treatment n = 97(%)	*p* value
Sex			
Male	29(46)	44(45.4)	
Female	34(54)	53(54.6)	0.93
Age	39.9 ± 12.0	42.9 ± 13.6	0.16
Co morbid			
DM	6(9.5)	14(14.4)	0.35
HTN	6(9.5)	16(16.5)	0.24
Duration of Asthma; years	16(6–25)	15(4.5–25.5)	0.90*
**symptoms**			
Weight loss	13(20.6)	22(22.7)	0.76
Dyspnea	37(58.7)	56(57.7)	0.90
Hemoptysis	22(34.9)	8(8.2)	<0.001
Pellet of sputum	14(22.2)	15(15.5)	0.27
Wheezing	15(23.8)	25(25.8)	0.77
Chest pain	18(28.6)	21(21.6)	0.31
Cough	51(81.0)	74(76.3)	0.48
Fever	15(23.8)	21(21.6)	0.74
Chest X-ray			
Unilateral	9(14.3)	16(16.5)	
Bilateral	54(85.7)	81(83.5)	0.70
Consolidation	12(19)	16(16.5)	0.67
Nodules	16(25.4)	28(28.9)	0.63
Cavitation	12(19)	9(9.3)	0.29
Fleeting Infiltrates	15(24)	22(23)	0.15
Fibrosis	10(16)	12(12)	0.29

ATT: Anti tuberculous therapy *Mann-whitney *U* test.

Out of 167, 27 (16.16%) patients of ABPA developed long term complications and 17 (62.96%) patients were in smear negative TB treatment group. Recurrent hemoptysis was developed in 7, chronic respiratory failure in 6, chronic pulmonary aspergillosis in 5 and cor-pulmonale in 5 patients.

## 5 Discussion

*Aspergillus* infection can lead to invasive and non-invasive diseases [9]. However, ABPA is a condition characterized by a hypersensitivity response to the fungus *Aspergillus* (most commonly *Aspergillus fumigatus*). Patient may also misdiagnosed as chronic persistent asthma [10].To our best of knowledge, this is the first study conducted in Pakistan on the misdiagnosis of ABPA with smear negative TB. Our study clearly shows that APBA is still under recognized in the country. Empiric ATT use is common and 82.5% patients had received ATT once.

Our results are in line with similar studies in other TB endemic countries [[11], [12], [13], [14]]. Due to the similar clinical and radiological features of both diseases patients with ABPA are often misdiagnosed as pulmonary TB in high endemic areas [11]. Studies showed that almost one third of ABPA patients in India are mistakenly diagnosed as pulmonary TB [12]. A 2006 study in India showed that out of 126 patients presenting to a chest clinic with ABPA, 59 were initially misdiagnosed as pulmonary TB and received ATT [12]. Similarly, a 2009 retrospective study revealed that 91% of patients with ABPA were initially diagnosed with pulmonary TB and were prescribed ATT [14]. In a 2019 study in China, out of 50 patients with ABPA, 5 were initially diagnosed with Pulmonary TB and received ATT therapy [15].

There is no one set method for diagnosing ABPA which is why it often becomes a diagnostic challenge [16]. The diagnosis is usually made following Rosenberg and Patterson's criteria though patients may be monitored for several years before the entire diagnostic criteria is met [17]. Central bronchiectasis with normal peripheral bronchi is considered to be a key diagnostic finding in ABPA [18]. Currently high-resolution CT (HRCT) chest is the modality of choice for identifying bronchiectasis [19]. Unfortunately HRCT chest by itself has a poor specificity in distinguishing between different causes of bronchiectasis [20]. Our study did not find a significant association regarding radiological findings in both groups, highlighting the limitations of radiology in diagnosing ABPA.

If untreated, ABPA may progress to irreversible bronchi damage and lung fibrosis along with hemoptysis, chronic sputum production, pneumonias and ultimately respiratory failure [21]. In our study 27 patients developed such complications, 17 were belonged to the smear negative group treated with ATT therapy. Complications such as chronic pulmonary aspergillosis and respiratory failure were almost equally found in both groups of patients. There was a significant association between hemoptysis and the smear negative TB group treated with ATT therapy. This finding can be explained by the progressive lung damage that occurs in ABPA patients who are misdiagnosed as pulmonary TB and are not appropriately treated for ABPA.

Ideally, an acid-fast bacillus smear test can help distinguish between TB and ABPA, but the real challenge arises in ruling out ABPA in patients who are smear and Gene xpert negative for TB. The sensitivity of the GeneXpert in smear negative HIV patient was as high as 98% with the specificity of 52% [22]. While the pooled sensitivity and specificity for smear-negative TB documented up to 67% and 98% for GeneXpert, 73% and 91% for Microscopic Observation Drug Susceptibility assay (MODS) [23].Why such a large number were misdiagnosed despite role out of GeneXpert in the country? This may be because of lack of ABPA knowledge and delay in referral to chest specialist. This clearly elucidates the importance of an early diagnosis and awareness regarding of ABPA.

This study highlights that ABPA quite often misdiagnosed with TB and treated with empirical ATT in high TB burden country like Pakistan. However our study have few limitations. Since it is a retrospective study, we were unable to standardize the diagnostic and treatment approaches in the two set of groups. Our study is a single center study. Therefore, out study can be used as a baseline for larger multicenter studies which can ensure proper surveillance of ABPA and thus better control of the disease.

## 6 Conclusion

In summary, ABPA is an under recognized entity in Pakistan. It is often mistakenly diagnosed as pulmonary TB due to sharing similar clinical and radiological features. A prolonged diagnosis of ABPA can cause progressive lung damage which can lead to fibrosis and respiratory failure. Greater physician awareness and implementation of appropriate diagnostic criteria is essential for a timely diagnosis of ABPA.

## Ethics approval and consent to participate

The study was approved by the ethical review committee (ERC) of Aga Khan University (AKU).

## Funding

None.

## Authors' contributions

**Nousheen Iqbal (NI)** has made contributions to conception and design, interpretation of data, drafting the manuscript and revising it critically for important intellectual content.

**Muhammad Dawood Amir Sheikh (MDAS)** has made contributions in interpretation of data, drafting the manuscript and revising it critically for important intellectual content.

**Kauser Jabeen (KJ)** has made contributions in conception and design, interpretation of data, drafting the manuscript and revising it critically for important intellectual content.

**Safia Awan (SA)** has made contributions in, interpretation of data, drafting the manuscript and revising it critically for important intellectual content.

**Muhammad Irfan (MI)** has made contributions to conception and design, interpretation of data, drafting the manuscript and revising it critically for important intellectual content.

All authors read and approved the final manuscript.

## Consent for publication

Not applicable.

## Availability of data and materials

The datasets used and/or analyzed during the current study are available from the corresponding author on reasonable request.

## Guarantor

The Guarantor is the one or more people who accept full responsibility for the work and/or the conduct of the study, had access to the data, and controlled the decision to publish.

All Authors have the data access and control the decision to publish.

## Consent

Studies on patients or volunteers require ethics committee approval and fully informed written consent which should be documented in the paper.

Authors must obtain written and signed consent to publish a case report from the patient (or, where applicable, the patient's guardian or next of kin) prior to submission. We ask Authors to confirm as part of the submission process that such consent has been obtained, and the manuscript must include a statement to this effect in a consent section at the end of the manuscript, as follows: "Written informed consent was obtained from the patient for publication of this case report and accompanying images. A copy of the written consent is available for review by the Editor-in-Chief of this journal on request”.

Patients have a right to privacy. Patients’ and volunteers' names, initials, or hospital numbers should not be used. Images of patients or volunteers should not be used unless the information is essential for scientific purposes and explicit permission has been given as part of the consent. If such consent is made subject to any conditions, the Editor in Chief must be made aware of all such conditions.

Even where consent has been given, identifying details should be omitted if they are not essential. If identifying characteristics are altered to protect anonymity, such as in genetic pedigrees, authors should provide assurance that alterations do not distort scientific meaning and editors should so note.

Not Required.

## Declaration of competing interest

None to declare.
